# Coordinated program between primary care and sleep unit for the management of obstructive sleep apnea

**DOI:** 10.1038/s41533-019-0151-9

**Published:** 2019-11-08

**Authors:** Mercè Mayos, Patricia Peñacoba, Anna María Pedro Pijoan, Carme Santiveri, Xavier Flor, Joan Juvanteny, Gabriel Sampol, Patricia Lloberes, José Ignacio Aoiz, Joan Bayó, Núria Grau, Ana M. Fortuna, Vicente Plaza, M. Antònia Llauger

**Affiliations:** 1 Sleep Unit, Department of Respiratory Diseases, Hospital de la Santa Creu i Sant Pau, Universitat Autònoma de Barcelona, Barcelona, Spain; 2 Centro de Investigación Biomédica en Red Enfermedades Respiratorias (CIBERES) (CB06/06), Instituto de Investigación Carlos III, Madrid, Spain; 3 Department of Medicine, Universitat Autònoma de Barcelona, Barcelona, Spain; 4 Àrea Bàsica de Salut (ABS) Gaudí, Institut Catalá de la Salut, Barcelona, Spain; 5 Service of Pneumology, Hospital Dos de Maig, Barcelona, Spain; 6 Centre d’Atenció Primària (CAP) Chafarinas, Barcelona, Spain; 7 Centre d’Atenció Primària (CAP) Trinitat Vella, Barcelona, Spain; 8 Service of Pneumology, Hospital Universitari Vall d’Hebron, Universitat Autònoma de Barcelona, Barcelona, Spain; 9 Centre d’Atenció Primària(CAO) El Clot, Barcelona, Spain; 10 Service of Pneumology, Hospital del Mar, Universitat Autònoma de Barcelona, Barcelona, Spain; 11 Centre d’Atenció Primària (CAP) Els Encants, Barcelona, Spain

**Keywords:** Respiratory tract diseases, Diagnosis

## Abstract

The purpose of this study is to develop and validate a work model in the primary health-care setting for identifying patients with obstructive sleep apnea–hypopnea syndrome (OSAHS) based on clinical variables and an ambulatory sleep monitoring study. After screening, patients with mild–moderate OSAHS could be managed by primary care physicians, whereas those identified with severe OSAHS would be referred to specialists from sleep units for starting specific treatment. The proposed model does not move the entire health-care process to a generally overburdened primary care level and favors the coordinated work and the necessary flexibility to adapt the model to challenges and perspectives of OSAHS.

## Introduction

Obstructive sleep apnea–hypopnea syndrome (OSAHS) is a common breathing disorder in the general population,^1^ with major clinical and socioeconomic consequences.^2^ OSAHS is characterized by repetitive episodes of upper airway collapse during sleep, resulting in cyclic decreases of arterial oxygen saturation and transient cortical arousals, leading to non-restorative sleep. Daytime sleepiness is a cardinal symptom of OSAHS and if left untreated, it leads to cognitive dysfunction, decrements in health-related quality of life, impaired work performance, and increased risk for accidents in the workplace and traffic accidents.^3,4^ Also, repetitive episodes of hypoxia and enhanced sympathetic activity trigger pathogenic pathways related to an increased risk for cardiovascular and metabolic morbidity^5–8^ and mortality.^9,10^

Nasal continuous positive airway pressure (CPAP) during sleep is the leading therapy for OSAHS.^11^ CPAP is highly effective in controlling obstructive respiratory events, improvement of symptoms and quality of life of patients affected with OSHAS,^12^ and significant reduction of blood pressure^13,14^ and insulin resistance.^15^ Treatment with CPAP has been associated with a decrease of all-cause and cardiovascular mortality.^9^

It has been estimated that the total cost of OSAHS would be higher than that of other chronic diseases such as asthma and chronic obstructive pulmonary disease (COPD) and similar to diabetes-related expenses.^16^ A high percentage of OSAHS-related costs are derived from societal consequences, including decreased work productivity and the burden of road traffic accidents.^17^

OSAHS is clearly underdiagnosed, which is largely attributed to oversaturation of sleep units where management of the disease has been focused so far and where there are long lists of patients waiting for diagnosis and treatment.^18^ Currently, there is consensus that a disease with such relevant impact on the individual’s health should involve all levels of care, with primary care taking a more active role in the diagnosis and even management of OSAHS.^19,20^ Different models of integral management of patients with high clinical probability of OSAHS in the primary care setting have been proposed, with non-inferior mid-term results to those reported in sleep units.^21–24^ However, although these results open new perspectives in management of OSAHS, the feasibility of the implementation of these models in clinical practice has been questioned. Among other limitations, it has been argued that primary care physicians have to assume a central role in the care of patients with OSAHS, which simplifies excessively the management of patients with an increasingly complex disease.^25^

## Aims

The PASHOS project (PASHOS is the Spanish acronym of Advanced Platform for Sleep Apnea Syndrome Assessment) is conceived with a different approach to the problem, that is, the implementation of a comprehensive inter-level coordinated program between primary care and specialist sleep centers for patients with OSAHS. Advantages of the program include screening and tentatively management of OSAHS in the primary care setting and prioritization of primary care referrals to specialized care for starting CPAP treatment. The main objective is to develop a simplified two-stage model for the management of patients with OSAHS in the primary care setting based on a screening questionnaire followed by an ambulatory sleep monitoring study at home, and based on that information, a decision to refer to a sleep unit or to manage them in the same primary care setting. The secondary objectives are as follows: (1) to assess the validity of management decisions made by primary care physicians (indication of CPAP versus no indication of CPAP), based on results of clinical evaluation and ambulatory sleep study as compared to therapeutic decisions made by specialists at the sleep unit; and (2) to analyze the cost-effectiveness of the coordinated strategy versus the strategy that includes assessment of OSAHS solely in the sleep unit.

## Discussion

This prospective and multicenter study protocol aims to validate a coordinating work model between primary care and the sleep unit for the management of OSAHS. The model includes training of reference health-care professionals from primary care and validation of simple screening tools that allow the family physician to select the patients who can be managed in the primary care setting (mild–moderate OSAHS) from those who have to be referred to sleep units to start specific treatment. The study differs from other interesting recent clinical trials^22–24^ in that it may be applicable to the entire phenotypic spectrum of patients, because inclusion is not restricted to a certain “a priori” clinical probability of OSAHS, which accounts for only 30% of the total OSAHS population.^26,27^ On the other hand, the model does not move the entire health-care process to a generally overburdened primary care level and favors the coordinated work and the necessary flexibility to adapt the model to challenges and perspectives of OSAHS.^28,29^

The exclusion of patients with moderate-to-severe COPD deserves a comment. Patients with overlap syndrome (COPD/OSAHS) present differential clinical characteristics than OSAHS patients without COPD. In these patients, screening questionnaires (e.g., Epworth sleepiness scale, STOP-Bang sleep questionnaire) or other clinical parameters are not well predictors of OSAHS, and it has been suggested that specific tools are necessary to evaluate the risk of OSAHS in this population.^30^ Moreover, poor sleep quality in patients with moderate-to-severe COPD may affect the diagnostic reliability of the simplified ambulatory sleep study. They also may present nocturnal hypoxemia due to multiple mechanisms, besides upper airway occlusion characteristic of OSAHS, such as ventilation/perfusion alterations or hypoventilation. It is therefore a complex sleep respiratory disorder that should be preferably studied with a full polysomnography.^31^

Limitations of the study include the upper age range of 75 years as an inclusion criterion, so that it would be necessary to validate the model in older age groups. Polysomnography was not used as the standard diagnostic test for the diagnosis of OSA in adult patients. Although respiratory polygraphy can underestimate the diagnosis of OSA, we adapt our model to common available resources in routine clinical practice and to recommendations of guidelines suggesting the use of respiratory polygraphy only in selected patients.^32^

In summary, we believe that results of this project could represent a relevant change in the different levels of care for the management of patients with OSAHS, probably applicable to different health-care systems.

## Methods

### Design and participants

This is a prospective multicenter study that includes the participation of sleep units from three tertiary care teaching hospitals in the urban area of Barcelona (Spain), a sleep unit of a secondary care hospital (with capacity to perform respiratory polygraphy studies and refer to a tertiary center if polysomnography is needed), and six primary care teams of the reference area of the four hospitals. The inclusion and exclusion criteria are shown in Table 1.

**Table 1:** Inclusion/exclusion criteria

Inclusion criteria	Exclusion criteria
• Men and women	• Cognitive impairment or psychosocial inability to perform the ambulatory sleep study
• Age ≥18 and ≤75 years	• Unstable or acute cardiovascular or cerebrovascular disease
• Patients visited at the participating primary care centers for any reason will be consecutively included according to a randomization schedule to reach the necessary sample size	• Chronic insomnia (<5 h of sleep/day)
	• Previous diagnosis of OSAHS
	• Relevant respiratory comorbidity that may interfere with arterial blood saturation measurements
	• Moderate-to-severe COPD (FEV_1_/FVC < 0.7%, FEV_1_ < 50% predicted)
	• History of neuromuscular disease
	• Patient’s refusal to participate in the study

*OSAHS* obstructive sleep apnea hypopnea syndrome, *COPD* chronic obstructive pulmonary disease, *FEV*_*1*_ forced expiratory volume in one second, *FVC* forced vital capacity

This research has been already approved by the Clinical Research Ethics Committee of the 10 participating centers. Written informed consent will be obtained from all patients. The study has been registered at ClinicalTrials.gov (identifier NCT02591979).

### Training protocol

Prior to the beginning of the study, the following training program was carried out:

- Training of two reference professionals, one primary care physician, and a nurse for each of the primary health-care centers participating in the study.
- Theoretical training (4 h) including updated information of definition and epidemiology of OSAHS, clinical presentation and management of specific questionnaires, clinical impact of the disease, diagnosis of OSAHS, treatment and prevention, and aspects related to the care of patients in the framework of the local health-care system.
- Practical training for nursing (3 days, 6 h/day) including management of home diagnostic equipment and interpretation of data, as well as basic training in more complex sleep studies.
- Training for primary care physicians (3 days, 6 h/day) including indication and interpretation of the different diagnostic sleep studies and clinical management of the patient with OSAHS in the primary care setting.

At the end of the training period, all doctors (primary care physicians and sleep specialists) analyzed 10 simulated cases of OSAHS with different levels of severity and registers with technical errors. The degree of agreement between each participating center (6 primary health-care centers, 4 sleep units) and the coordinating sleep unit that selected the simulation cases is shown in Table 2. The degree of agreement ranges from moderate (Cohen’s kappa coefficient [*κ*] 0.47) to perfect agreement (*κ* = 1.0). Concordance was higher in examples of extreme cases (OSAHS clearly present or clearly absent), whereas there was a greater variability in decision-making for cases with suspicion of mild-to-moderate OSAHS. Finally, therapeutic decisions simulation cases were reviewed in a subsequent joint meeting of all study participants.

**Table 2:** Agreement between each participating center and the coordinating center

Participating center	Cohen’s kappa (*κ*)	Asymptotic standard error^a^	Approx. *T*^b^	Approx. Sig.
Sleep unit #2	0.571	0.182	3.086	0.002
Sleep unit #3	1.0	0.0	5.331	0.000
Sleep unit #4	0.865	0.128	4.749	0.000
Primary care center #1	0.474	0.182	2.868	0.004
Primary care center #2	0.595	0.173	3.505	0.000
Primary care center #3	0.571	0.173	3.203	0.001
Primary care center #4	0.861	0.127	4.642	0.000
Primary care center #5	0.595	0.173	3.505	0.000
Primary care center #6	0.459	0.185	2.642	0.008

Coordinating center: sleep unit #1

^a^Not assuming the null hypothesis

^b^Using the asymptomatic standard error assuming the null hypothesis

### Study variables and data collection

The following variables will be collected: anthropometric (weight, height, body mass index) and sociodemographic (age, sex) data; history of cardiovascular diseases and cardiovascular risk factors; other comorbidities, including cerebrovascular, metabolic, neurologic, respiratory, and psychiatric diseases; and pharmacological treatment. Clinical history directed to assessment of sleep breathing disorders, including a specific clinical questionnaire of suggestive symptoms of OSAHS, the Berlin questionnaire,^33^ the STOP-Bang sleep apnea questionnaire,^34^ and daytime sleepiness using the Epworth sleepiness scale.^35^ Other studies include forced spirometry^36^ and conventional polysomnography/respiratory polygraphy^37,38^ recording the following data: apnea–hypopnea index (AHI), obstructive apnea index (episodes/h), hypopnea index (episodes/h), central apnea index (episodes/h), mixed apnea index (episodes/h), mean peripheral oxygen saturation (SpO_2_), time spent at SaO_2_ < 90% (CT90), falls in SpO_2_ ≥ 3% (oxygen desaturation index [ODI]-3%) and ≥ 4% (ODI-4%) per hour of recording, and time spent in the supine position.

The ambulatory monitoring sleep study will be carried out at the patient’s home using a Bitmed Sleep&Go polygraph (Sibelmed, Barcelona, Spain). The reference primary care nurse will be in charge of the analysis of sleep quality, discarding the periods of deficient signal but will not analyze all the events manually. The minimal valid recording time is defined as 5 h. Polygraphy may be repeated if a poor signal acquisition is detected or the patient reports poor sleep quality on the night of the study. The following variables will be recorded: mean SpO_2_, ODI-3%, ODI-4%, AHI (cut-off points 5–15 for mild OSAHS, >15–<30 for moderate OSAHS, and ≥30 for severe OSAHS), and percentage of time in supine position.

A file transfer protocol (FTP) has been developed for transmitting sleep recorded data from primary care centers to the corresponding reference sleep units. The FileZilla® software (version 3.3.0.1 - GNU General Public License, version 2, June 1991, Free Software Foundation Inc.) is the server that supports FTP. The program has been installed in each participating center. A username and password was created for each research team, giving access to its folder only. Primary care teams can only upload files to their folders, whereas physicians from the sleep units can only download files from primary care centers of their reference area. The coordinating center has full access to folders of all participating centers in order to review and solve any possible incidents that may happen. All patients’ data will be anonymized.

### Model design and validation protocols

In all patients who fulfilled the inclusion criteria (Table 1), data on anthropometric variables, clinical variables, and specific study questionnaires (Berlin questionnaire, STOP-Bang sleep apnea questionnaire, and Epworth sleepiness scale) will be collected at the primary care setting. Patients with a high probability of OSAHS according to results of the Berlin questionnaire and 1 out of 3 patients with a low probability of OSAHS will undergo the ambulatory monitoring sleep study and will be referred to the sleep unit for completion of OSAHS evaluation. The selection of 1 out of 3 patients with low probability of OSAHS is established in order to balance the study sample and tries to achieve a similar final proportion of patients with high and low probability of OSAHS, taking into account that the expected OSAHS population is approximately 30%.

On the basis of clinical data and results of ambulatory sleep study, the primary care physician will take a clinical decision according to the following four diagnostic–therapeutic scenarios: (a) low suspicion of OSAHS and no need of treatment; (b) mild/moderate suspicion of OSAHS and conservative treatment; (c) suspicion of OSAHS and candidate for CPAP treatment; and (d) indeterminate in the presence of low quality of ambulatory sleep study recording or discrepancy between clinical data and results of ambulatory sleep study.

**Fig. 1:**
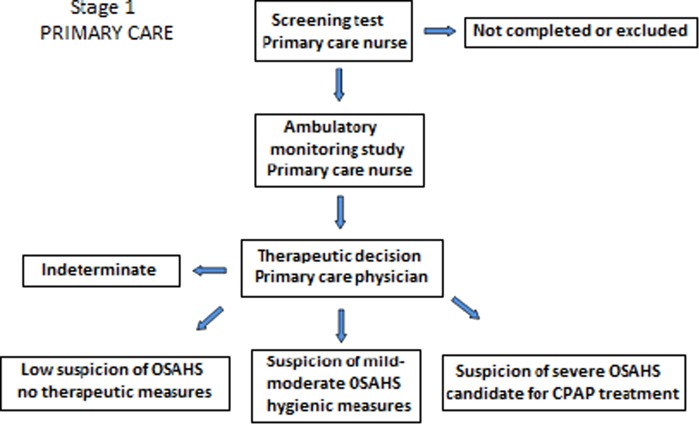
Steps involving validation of the model in primary care

**Fig. 2:**
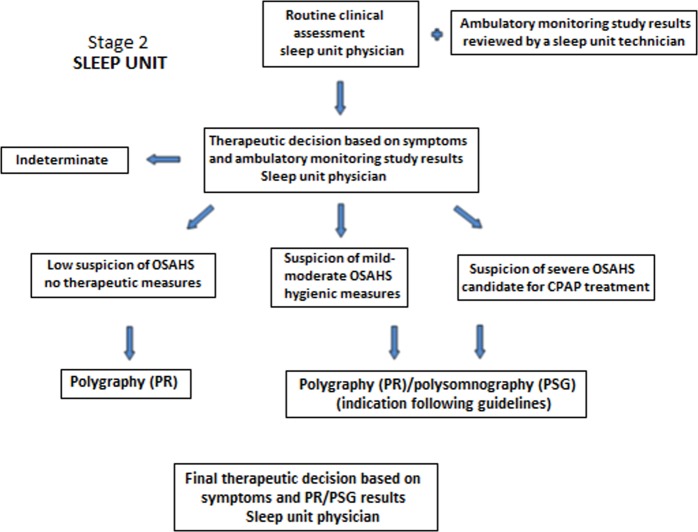
Steps involving validation of the model in specialized care (sleep units)

All patients undergoing home sleep monitoring independently of the high or low initial clinical probability of OSAHS will be referred to the sleep unit, where specialists, in a blinded fashion regarding primary care results and with all documentation available, will take a further diagnostic–therapeutic decision according to the same four possible scenarios defined in primary care. Finally, all patients referred to the sleep unit will undergo a complete respiratory polygraphy or conventional polysomnography at the sleep unit to establish a definite diagnosis and therapeutic indication. This approach will allow analyzing the diagnostic and therapeutic concordance between primary care and specialized care, using the same basic tools (clinical findings and ambulatory sleep monitoring) and with the final results of the gold standard examination. The two stages of the model are shown in Figs 1 and 2.

### Sample size calculation

The sample size has been calculated considering a minimal prevalence of OSAHS of 25% in the population attended in the primary care setting. Assuming a loss of 15% at follow-up, an alpha error of 5%, and a 90% statistical power, a total sample of 198 patients would be required for 90% sensitivity in the validation sample (99 patients in each arm with low/high probability of OSAHS). However, the number of valid initial questionnaires should be much higher, given that all patients with a priori high probability of OSAHS and 1:3 of those with low probability will be included. In this way, it is intended to adjust the performance of sleep studies and the balance between OSAHS subjects/non-OSAHS subjects. Therefore, a minimum number of 396 valid questionnaires are planned.

### Statistical analysis

Categorical variables will be expressed as frequencies and percentages and quantitative variables as mean and standard deviation (SD), median and interquartile range (25th–75th percentile), and 95% confidence interval (CI). Bivariate analysis will include chi-square (χ^2^) test or Fisher’s exact probability test for categorical data and Student’s *t* test or Mann–Whitney *U* test for continuous data according to the conditions of application. The degree of agreement between the participating centers regarding ten simulated cases has been analyzed with the Cohen’s kappa correlation coefficient (Table 2). The clinical prediction model will be based on logistic regression analysis. All variables statistically significant in the bivariate analysis and those considered to be clinically relevant will be included in the logistic regression model. Hosmer–Lemeshow goodness-of-fit test and the area under the receiver operating characteristics curve will be used to validate the model. Statistical significance is set a *P* < 0.05 (two tailed). Cost analysis will be performed from the Spanish Health System perspective using a bottom–up costing approach. Direct costs of tests (ambulatory sleep monitoring study, polygraphy, polysomnography), personnel, amortization of equipments, transfer of patients, and number of visits will be considered. For the cost-effectiveness analysis, false positive and false positive cases of the coordinated strategy versus conventional management will be considered. A Markov model will be constructed following the methodology proposed by Pietzsch et al.^39^ in which, with a time horizon of 10 years, the effect of treatment of OSAHS will be considered according to data collected from the literature on health-related quality of life, cardiovascular comorbidity, and risk of traffic accidentability.

## Supplementary information

PASHOS protocol

Reporting Sum
