# Supplementary material for: Coordinated program between primary care and sleep unit for the management of obstructive sleep apnea
Source: NPJ Prim Care Respir Med. 2019 Nov 8;29:39. doi: 10.1038/s41533-019-0151-9 (PMC6841945; doi:10.1038/s41533-019-0151-9)
Supplement: Supplementary file 1 — PASHOS protocol [file 41533_2019_151_MOESM1_ESM.pdf]

PASHOS Project: Advanced Platform for Obstructive Sleep Apnea (OSA) Assessment:

A Multi-Centre Study for the Validation of a OSA Screening Model in Primary Care

SUMMARY OF THE PROJECT PROPOSAL:

Study General Objective:

The purpose of this study is to develop and validate a work-model for Primary Care for identifying patients with Obstructive Sleep Apnea Syndrome (OSA), based on clinical variables and an ambulatory monitoring study.

Study Specific Objectives:

.- To establish the validity of an Ambulatory Monitoring Study (AMS) for identifying patients with OSA, compare it with Sleep Unit studies, Polygraphy (RP) and Polysomnography (PSG), and create a screening model for the detection of OSA in Primary Care.

.- To analyze the validity of the therapeutic decisions (CPAP) made by Primary Caregivers, in comparison with the therapeutic decisions made by Sleep Unit specialists.

.- To analyze the cost-effectiveness of AMS.

Methods:

Prospective multicenter study. Patients aged between 18-75 years visited at Primary Care consultations will be included according to a randomization schedule to reach the sample size (198). The study involves 4 Sleep Lab Units and 6 Primary Care Centers in Barcelona. Two models of evaluation will be used: one in the Primary Care center and the other in the Sleep Lab Units. **Outcomes**: anthropometric data, cardiovascular history and risk factors, self-assessed questionnaire of sleep disorders, ambulatory monitoring study data and polygraphic/polysomnographic data. **Statistical analysis**: Calculation of the area under the curve (AUC) of the ROC curve (Receiver Operating Characteristics). The best clinical prediction model will be detected by using logistic regression. Variables with clinical significance in the bivariate analysis and variables considered clinically relevant will be included. The resulting model will be validated with the goodness-of-fit test (Hosmer and Lemeshow test). **Cost analysis**: Methodology according to Pietzoch JB et al. Sleep 2011; 34: 695-709.

DETAILED DESCRIPTION OF THE PLANNED PROGRAM INCLUDING MILESTONE AND TIME PLANNING

Background

Obstructive Sleep Apnea (OSA) is a highly prevalent disorder in the general population (1). It is associated with an impaired quality of life (2) and an increased work and traffic accident risk (3). It is also associated with high blood pressure (4), diabetes mellitus (5), cardiovascular and cerebrovascular diseases and increased mortality (6).

OSA is characterized by repeated episodes of airflow cessation that cause cyclic falls of arterial oxygen saturation and arousals. These consequences decrease sleep quality and result in non-refreshing sleep. Daytime sleepiness is one of the main symptoms of patients with OSA and produces impaired cognitive functions, lack of concentration, loss of memory, coordination difficulties and impaired quality of life. Repeated episodes of hypoxia-reoxygenation and an increase of sympathetic activity can stimulate different pathogenic pathways such as oxidative stress, endothelial dysfunction, hypercoagulability and insulin resistance causing atherogenesis. These disturbances create a greater risk of cardiovascular and metabolic diseases (7).

The severity of this syndrome is defined by the apnea-hypopnea index (AHI). The AHI is obtained by dividing the apnea (total occlusion) and hypopnea (partial occlusion) number by the hours of sleep. An AHI > 15 is associated with increased cardiovascular risk, with or without symptoms, and this risk is greater at AHI > 30 (8).

It is estimated that between 3-6% of the Spanish population present this syndrome. This prevalence increases to 24-25% in cases of a high AHI without symptoms (1). In some risk-populations the presence of OSA is very high. About 30% of patients with arterial hypertension have OSA (9). This figure increases to 80% in patients with hypertension refractory to medical therapy (10).

Our research group has recently shown a prevalence of 72% in morbidly obese candidates for bariatric surgery (11).

Patients with OSA double their use of sanitary resources during the ten years before diagnosis, including hospitalizations. A high percentage of the cost of this syndrome arises from other social consequences such as poor work performance and traffic accidents (12). In the U.S.A. the estimated total cost of OSA is greater than the outlay for other chronic diseases such as asthma and COPD and equals the outlay for diabetes mellitus (13).

Basic treatment consists of applying continuous positive airway pressure (CPAP) during sleep. CPAP is an effective treatment that corrects obstructive respiratory disturbances and improves symptoms and quality of life.

In different randomized, placebo controlled trials, CPAP has demonstrated a significant reduction in hypertension and an improvement of insulin resistance (14). In patients with severe OSA, CPAP treatment decreases mortality due to cardiovascular disease (6).

The guidelines establish the use of CPAP depending on the severity of the disorder, its impact on the quality of life and its possible association with cardiovascular, cerebrovascular or respiratory diseases. In general, CPAP is indicated in patients with severe OSA and in those with moderate OSA with relevant symptoms or comorbidity (8,15).

Although the number of Sleep Units has increased considerably over the last few years, available resources are still insufficient for all the demand for care generated by OSA (16). In our public-health area (Barcelona Eixample Dreta), the delay time estimated for a screening study is 6 months. If a study suggests the presence of a respiratory sleep disorder, the delay time for a diagnostic study increases by an additional 6 months.

The diagnosis of OSA is based on a sleep study that detects different disturbances (apneas, hypopneas and episodes of airflow limitation) and their immediate consequences (arterial desaturation, arousals, heart rhythm disorders, etc). Conventional polysomnografy (PSG), monitored by a technician in the sleep laboratory, is the gold standard diagnostic test for suspected OSA. PSG is an expensive, laborious and technically complex technique and is not available at all centers. To address these difficulties, portable devices that record only respiratory variables have been developed, such as respiratory polygraphy (RP). RP is a diagnosis validated method in a high percentage of patients (8.13). It is easier to interpret than PSG but also requires training and experience in signals analysis.

Recently, even simpler ambulatory monitoring devices have appeared on the market. They are extremely user-friendly, in terms of both use and analysis. These tools can play an important role in disease detection outside hospital Sleep Units, especially for at-risk populations and patients with serious illness.

Although these devices are designed to be used by non-expert personnel, the validation studies existing until now have been conducted in specialized Sleep Units (17). These systems are therefore not being used at the healthcare level for which they were designed initially.

Currently, the Primary Care physician (PCP) usually refers all patients with suspected OSA to the specialist, without considering clinical probability (high or low) or comorbidity. The prevalence of symptoms related to OSA in patients visited for any reason by PCPs is far higher than for the general population (same age and gender) (18). That is because the population attended at Primary Care centers has specific characteristics: being more obese and with more comorbidities. Additionally, several series have shown that PCPs have a surprisingly low level of suspicion of this disease, even in clearly at-risk populations (19). For example, in the city of Barcelona, the diagnosis of OSA is listed in only 1.49% of the computerized medical records. Specifically, in the “Dreta de l'Eixample”, one of the areas where this project will be carried out, the diagnosis of OSA appears in only 1.38% of the records.

These results can be explained by lack of information about this relatively recent disease, the poor relationship between Sleep Units and Primary Care, and the difficulty in identifying symptoms suggestive of OSA.

The immediate future of the management of OSA requires coordination between all levels of healthcare: Sleep Units at hospitals, less complex units and Primary Care centers. It also requires the inclusion of simple screening tools, outside hospitals, for detecting patients requiring priority attention. In this regard, outside our country, the results of some experiences with very promising results have recently been published. Chai-Ch L Coetzer et al. (20) validated a screening model in 150 patients in Primary Care centers. This model included clinical variables and a home overnight oximetry study. The diagnostic profitability of the model showed a sensitivity of 0.88 (95% CI from 0.60 to 0.98) and a specificity of 0.82 (95% CI 0.70-0.90). It must be taken into account that these were consecutive patients visited in Primary Care centers, without a previous clinical suspicion of OSA. The same research group analysed the clinical efficacy and costs of an integral model for patients with OSA at Primary Care level, including therapeutic management (21). The study showed comparable results in terms of clinical and cost outcomes at Primary Care level versus specialized care. Until now, similar projects have not been carried out in our country.

Bibliography

1.- Durán J, Esnaola S, Ramón R, Iztueta A. Obstructive sleep apnea-hypopnea and related clinical features in a population-based sample of subjects aged 30 to 70 years. Am J Respir Crit Care Med 2001;163:685-689.

2.- Engleman HM, Douglas NJ. Sleepiness, cognitive function and quality of life in obstructive sleep apnoea/hypopnoea síndrome. Thorax 2004;59:618-622.

3.- Terán-Santos J, Jiménez-Gómez A, Cordero-Guevara J, and the Cooperative Group Burgos–Santander. The association between sleep apnea and the risk of traffic accidents. N Engl J Med 1999;340:847–851.

4.- Peppard PE, Young T, Palta M, Skatrud J. Prospective study of the association between sleep-disordered breathing and hypertension. New Eng J Med 2000;342:1378-1384.

5.- Levi O, Bonsignore MR, Eckel J. Sleep-disordered breathing and metabolic consequences. Eur Respir J 2009;39:243-60.

6.- Marin JM, Carrizo SJ, Vicente E, Agusti AG. Long-term cardiovascular outcomes in mes with obstructive sleep-apnoea-hypopnoea with or without tratment with continuous positive airway pressure: an observational study. Lancet 2005;365:1046-53.

7.- Ryan S, Taylor CT, McNicholas WT. Selective activation of inflammatory pathways by intermittent hypoxia in obstructive sleep apnea syndrome. Circulation. 2005;112:2660–7.

8.- Lloberes P, Durán-Cantolla J, Martínez-García MÁ, Marín JM, Ferrer A, Corral J et al. Diagnosis and treatment of sleep apnea-hypopnea syndrome. Spanish Society of Pulmonology and Thoracic Surgery. Arch Bronconeumol. 2011;47:143-56.

9.- Hedner J, Bengtsson-Bostrom K, Peker Y, Grote L, Rastam L, Lindblad U. Hypertension prevalence in obstructive sleep apnoea and sex: a population-based case-control study. Eur Respir J 2006;27:564–570.

10.- Logan AG, Perlikowski SM, A.Mente A, Tisler A, Tkacova R, Niroumand M, Leung RS, Bradley TD. High prevalence of unrecognized sleep apnoea in drug-resistant hypertension. J Hyperten 2001;19: 2271–2277.

11.- Gasa M, Salord N, Fortuna AM, Mayos M, Vilarrasa N, Dorca J, Monserrat JM, Bonsignore MR, Monasterio C. Obstructive sleep apnoea and metabolic impairment in severe obesity. Eur respir J 2011;38:1089-1097.

12.- Krieger MH, Ross L, Delaive K, Walld R, Horrocks J. Utilization of health care services in patients with severe obstructive sleep apnea. Sleep 1996;19:111-6.

13.- Alghanim N, Comondore VR, Fleetham J, Marra CA, Ayas NT. The economic impact of obstructive sleep apnea. Lung 2008;186:7-12

14.- Sharma SK, Agrawal S, Damodaran D, Sreenivas V, Kadhiravan T, Lakshmy R, et al. CPAP for the metabolic syndrome in patients with obstructive sleep apnea. N Engl J Med 2011;365:2277-2286.

15.- Epstein LJ, Kristo D, Strollo PJ Jr, Friedman N, Malhotra A, Patil SP et al. Adult Obstructive Sleep Apnea Task Force of the American Academy of Sleep Medicine. Clinical guideline for the evaluation, management and long-term care of obstructive sleep apnea in adults. J Clin Sleep Med 2009;5:263–276.

16.- Masa F, Barbé F, Capote F, Chiner E, Diaz de Atauri J, Durán J, et al. Recursos y demoras en el diagnóstico del syndrome de apneas-hipopneas durante el sueño (SAHS). Arch Bronconeumol 2007;43:188-98

17.- Oktay B, Rice TB, Atwood CW Jr, Passero M Jr, Gupta N, Givelber R et al. Evaluation of a single-channel portable monitor for the diagnosis of obstructive sleep apnea. J Clin Sleep Med. 2011 Aug 15;7(4):384-90. doi: 10.5664/JCSM.1196.

18.- Netzer NC, Hoegel JJ, Loube D, Netzer CM, Hay B, Alavarez-Sala R, et al. Prevalence of symptoms and risk of sleep apnea in primary care. Chest 2003;124:1406-1414.

19.- Broström A, Sunnergren O, Arestedt K, Johansson P, Ulander M, Riegel B, Svanborg E. Factors associated with undiagnosed obstructive sleep apnoea in hypertensive primary care patients Scand J Prim Health Care 2012;28:89-94.

20.- Chai-Coetzer Ch, Antic NA, Rowland LS, Catcheside PG, Esterman A, Reed RL, Williams H, Dunn S, McEvoy RD. A simplified model of screening questionnaire and home monitoring for obstructive sleep apnoea in primary care. Thorax 2011;66:213-219.

21.- Chai-Coetzer Ch, Antic NA, Rowland LS, Reed RL, Esterman A, Catcheside PG, Vowles N, Williams H, Dunn S, McEvoy RD. Primary care vs specialist sleep center management of obstructive sleep apnea and daytime sleepiness and quality of life. JAMA 2013;309(10):997-1004.

Rationale

The purpose of this project is to assess the efficacy of a sequential screening model in Primary Care centers for detecting and classifying patients with OSA according to severity. The clinical model used will, like recent studies published in the literature, be based on a set of clinical variables and the data obtained by a simplified monitoring device.

To achieve this, we have at our disposal three Sleep Units, attending an urban population of more than one million inhabitants and six Primary Care groups with different administrative and operational structures.

If the results are as expected, this screening model will prove to be a simple tool for screening for OSA at Primary Care level, thereby making it possible to avoid the referral of a large number of patients to Sleep Units. After a first analysis at Primary Care level, PCPs would be able to rule out OSA and, moreover, prioritize patients with severe disease.

Finally, we believe that the availability of this screening-model will have a positive effect on the impact of this disease and its process costs.

Hypothesis

It is possible to identify patients with OSA at Primary Care level with great diagnostic reliability by using a screening model that includes clinical variables and data provided by an ambulatory monitoring device.

The cost-effectiveness of this strategy, which includes the management of OSA at Primary Care level, is equal to or higher than the complete management carried out in the Sleep Unit.

General and specific objectives

General objective:

The main purpose of this project is to develop and validate a work-model for Primary Care for identifying patients with OSA, based on clinical variables and an ambulatory monitoring study.

Specific objectives:

- To establish the validity of an ambulatory monitoring device for identifying patients with OSA and to compare it with the RP / PSG performed at the Sleep Unit.

- To create a screening model for the detection of OSA.

- To analyze the validity of the treatment decision (CPAP vs. no CPAP) made by the PCPs, by using the ambulatory monitoring study, in relation to the decision made by the specialist Sleep Unit.

- To make an analysis of costs, including the cost-effectiveness of an outpatient strategy vs. a strategy including assessment at the Sleep Unit.

Methods

Study Design: Prospective and multicenter study.

Study population:

Patients visited in Primary Care centers will be consecutively included until the necessary sample size is reached. They will be selected according to a randomization schedule to avoid bias in the inclusion.

Inclusion Criteria:

- Patients between 18 and 75 years of age.

Exclusion Criteria:

- Cognitive impairment or psycho-physical deterioration that impede the performance of an ambulatory monitoring study.
- Worsened or unstable cardio-vascular or cerebro-vascular disease.
- Chronic insomnia (<5h sleep / day).
- Previous diagnosis of OSA.
- Relevant respiratory comorbidity that could interfere with arterial saturation measurements, such as moderate to severe chronic obstructive pulmonary disease (FEV1/FVC < 0,70 y FEV1 < 50% ref.v.).
- Neuromuscular disease.
- Refusal to participate in the study.

Field of study:

Three Sleep Units at three University Hospitals in Barcelona:

.- Hospital de la Santa Creu i Sant Pau (coordinator)

.- Hospital de la Vall d'Hebron

.- Hospital del Mar

.- Hospital Dos de Maig

Six Primary Care centers in the reference area of these three hospitals.

Outcome Measures

- Primary outcome: apnea-hypopnea index (AHI) obtained in Sleep Unit by PSG/RP study.

- Secondary outcomes:

Anthropometric and socio-demographic data: age, sex, weight, height, body mass index (BMI) and waist circumference.

Evaluation of medical history and cardiovascular risk factors: hypertension, diabetes, dyslipidemia, smoking, alcoholism, metabolic syndrome, heart disease (ischemic heart disease, arrhythmia, heart failure), cerebro-vascular disease (transient ischemic attack, ischemic or hemorrhagic) and peripheral vascular disease. Drug treatment.

Medical records focussed on Respiratory Sleep Disorders: specific clinical questionnaire on symptoms suggestive of OSA. Berlin-questionnaire in Spanish version. (Chest 2003;124:1406-1414).

Lung function tests: spirometry according to SEPAR recommendations. (Arch Bronconeumol 2013; 49: 388-401).

Self-perceived sleepiness analyzed by Epworth scale (MW Johns, Sleep 1991; 14: 540; validation in Castilian: Med Clin Ferrer M 1999.113: 250-255.).

Respiratory Polygraphy (RP) / Conventional Polysomnography (PSG):
Apnea-hypopnea index (IAH), obstructive apnea index, hypopnea index, central apnea index, mixed apnea index, sleep efficiency (total sleep time/recording time), percentage of time of phases NREM1, NREM2, NREM3 and REM, initial saturation, mean saturation (SO_2), minimum saturation and cumulative time percentage with saturation lower than 90% (CT90%).

Ambulatory monitoring study (AMS): AHI, mean saturation, 4% desaturation index (ID4%), 3% desaturation index (ID3%) and CT90%.

<u>Sample size</u>

The sample size has been calculated considering a minimum prevalence of 25% of OSA in the population attending Primary Care Medicine. Assuming 15% of losses to follow-up, an error of 5% and a power of 90%, a total sample of 198 patients was estimated as necessary to achieve a sensitivity of 90%.

According to the methodology proposed by Chai-Coetzer et al. (Thorax 2011; 66: 213-219), the screening study will be carried out taking into account clinical probability based on the Berlin Questionnaire (Netzer NC. Chest 2003; 124:1406-14). The study will be carried out in all patients with a high probability of OSA but in only 1 of 3 patients with a low probability. Therefore, a minimum of 396 valid questionnaires must be completed.

<u>Methodology</u>

1.- Training Time:

- Leader identification at each center: Primary Care nurses and physicians.

- Training Protocol, in charge of Sleep Units:

Theoretical Training: Four hours of theoretical training for the leaders participating in the project:

Definition and epidemiology of OSA

Symptoms and management

Clinical impact of the disease

Treatment and prevention

Assistance aspects

Practical Training:

Three days of practical training for nurses (6 hours per day): Management of ambulatory monitoring study and interpretation of the records. Basic training in more complex studies.

Three days of practical training for physicians (6 hours per day): Indication and interpretation of ambulatory monitoring studies. Outpatient clinical management.

2.- Design and Validation Time:

All patients eligible for inclusion in the study will perform the two models of validation (in the Primary Care center or in the Sleep Unit), as shown below (Stages 1 and 2):

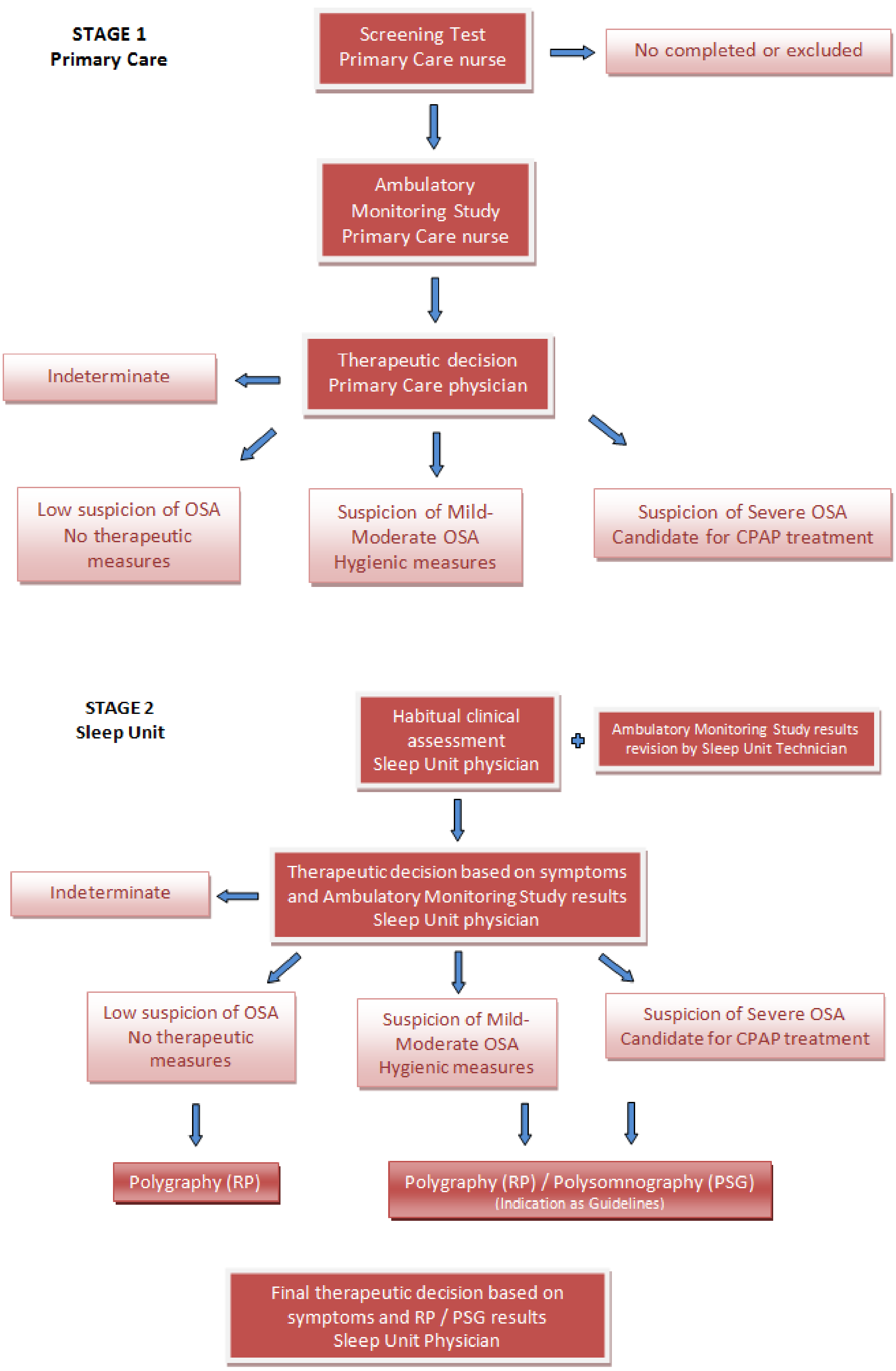

Ambulatory Monitoring Study (AMS):

The study will be performed in the patient's home with a Screen & GO device (BITMED, SibelGroup, Spain). The Primary Care nurse will record the anthropometric variables on the day before the test, will train the patient in the use of the device and will program it according to the usual sleep rhythm of the patient. To ensure the correct management of the equipment, a sheet with some instructions will be given to the patient.

The device will be returned to the Primary Care center the following morning. A brief questionnaire about the quality of sleep on the night of the study will be collected, estimating the start and end of the test. The nurse will be responsible for analyzing the quality of the study and will remove periods of poor signal but will not analyze the respiratory events manually: the recording time will be defined as the time passed from 15 minutes after sleep onset (according to previous questionnaire) to 10 minutes before completion. The minimum valid record-time will be 5 hours.

The analysis of the study will be done automatically by the device software. The following variables will be collected: mean SO2, ID4%, ID3%; CT90% and AHI.

The study could be repeated (only once) if a high percentage of poor signal acquisition is detected or if the patient reports poor quality of sleep during the night of the study.

To complete the assessment, a standard sleep study by RP / PSG will be performed, allowing for the final validation of the process.

The final treatment decision will always be made by the physician involved in the project, either in the Primary Care centre or in the Sleep Unit.

RP / conventional PSG: in every case, an overnight sleep study will be done in the Sleep Unit. Conventional PSG must include at least 6 channels: electroencephalography (EEG), electrooculography (EOG), mental electromyography (EMG), hemoglobin oxygen saturation detected by oximetry, oro-nasal airflow by thermistor and nasal-cannula, thoraco-abdominal movements by inductance plethysmography, electrocardiography (ECG), anterior-tibial electromyography and body position. RP will include every channel except EEG, EOG, EMG and ECG. The staging of sleep and respiratory events will be analyzed by the technician of the Sleep Unit, who will be blind to the results of the study at Primary Care level. The sleep study will be classified according to published guidelines (AASM 2007, SEPAR Manual procedures). The minimum recording time should be 6 hours and the minimum total sleep time, 3 hours.

The following data will be collected: Apnea-hypopnea index (AHI), obstructive apnea index, hypopnea index, central apnea index, mixed apnea index, sleep efficiency (total sleep

time/record time), percentage of time in phases NREM1, NREM2, NREM3 and REM, initial saturation, mean saturation (SO2), minimum saturation and cumulative time percentage with saturation lower than 90% (CT90%).

Data management and analysis

The main objective of this study is to obtain a sufficiently accurate calculation of the area under the curve (AUC) of the Receiver Operating Characteristics (ROC) curve. The expected sensitivity value is high, close to 90%. With this number of cases the accuracy of the AUC will not be less than 10%. In all cases, the interval confidence of calculated rates is assumed to be 95%.

A descriptive analysis will be performed using means, medians, confidence intervals, interquartile ranges for quantitative variables and table of frequencies for qualitative variables. In qualitative variables, the Chi-square test or Fisher exact test will be used for bivariate analysis. In the case of quantitative variables, the T-test or Mann-Whitney U test will be performed according to normal distribution.

Finally, logistic regression will be used to find the best clinical prediction model. Variables showing clinical significance in the bivariate analysis and those considered clinically relevant will be included. A goodness-of-fit test (Hosmer Lemeshow test) will be performed to validate the resulting model. The AUC of the ROC will be used to calibrate the model.

In all cases, the significance level will be, as usual, 5% (alpha = 0.05), bilateral approach.

We will use IBM-SPSS (V. 21.0) software to perform the analysis.

In the analysis of costs we will consider the direct costs of all the tests (ambulatory monitoring study, polygraphy or polysomnography), staffing costs, the amortization of the devices, the transfer of the patients and the number of visits. We will also take into account the false-positive and negative diagnosis (*Pietzoch JB et al. An integrated Health-Economic Analysis of Diagnostic and Therapeutic strategies in the treatment of moderate to severe obstructive sleep apnea. Sleep 2011;34: 695-709*).

Quality Control

Each investigator will ensure the accuracy and the integrity of the data and the reports. The data collected in the Data Collection Notebook, derived from source-documents, will be

concordant, and discrepancies should be justified. The research coordinator of the study will store the documents for at least 5 years after the completion. At the request of a monitor, auditor, Ethics Committee or Health Authority, the research coordinator will have available all the files related to the study. The coordinator will allow direct access to all the data and documents to perform monitoring, audit, a review by the Ethics Committee and test inspections by the competent authorities.

Limitations

- Limitations of RP: sleep time is not equivalent to study time and this can underestimate the values. To avoid this, we will take into account the quality of sleep provided by the patient. Thus, this aspect will not significantly limit the main objective: to differentiate patients with severe disease or mild disorders.

- Acceptance rate of participation: In a previous study with similar characteristics, the acceptance rate of participation was 40% of the survey respondents. Considering the large population usually attended at Primary Care centers in the urban area of Barcelona, this is not an expected limitation for reaching sample size. We must take into account that patients accepting their inclusion in the study could be those with greater comorbidity. This could give rise to a bias in the sample.

Time Planning

TASKS	Planning															
	2015				2016				2017				2018			
Project coordination meetings	■	■														
Primary Care staff training	■	■														
Recruitment Period			■	■	■	■	■	■								
End of study at Sleep Unit									■	■	■	■				
Results analysis													■			
Results publication														■	■	■

Considerations concerning patient information and consent form

The study will be conducted in strict accordance with international ethical guidelines for medical research in humans. Each investigator is responsible for ensuring that the study is conducted according to the standards set out in the Declaration of Helsinki.

Before starting the study, the Ethics Committee of each participating center must approve the study protocol, the information letter and the consent form.

Resources

The Sleep Unit at Hospital de la Santa Creu i Sant Pau, the coordinator of this study, has two Pulmonologists and two specialized nurses who are responsible for all respiratory diseases related to sleep. It also has two additional nurses who perform overnight sleep studies and CPAP titration. In this Unit, we perform about 1500 sleep studies per year, including outpatient studies, and attend 3,000 consultations related to OSA. There are four hospitalization beds, two PSG devices, 4 RPs, 2 single-channel devices and 3 CPAP titration devices.

The other two Sleep Units located in Hospital Vall d´Hebron (Multidisciplinary Sleep Unit) and Hospital del Mar, also have the necessary facilities and experience in the use of the techniques involved in this project. All of these procedures are part of their routine clinical practice.

All three hospitals have a great commitment to the transfer of part of this assessment to outpatient care medicine.

The Primary Care teams involved have the support of a variable number of physicians and nurses who are responsible for the first level of medical attendance. At the Primary Care level there are computerized medical records registering health problems, medical examinations (i.e. spirometric data), chronic-disease monitoring devices and diagnostic assistance. All the Primary Care centers have spirometers and trained nurses. The Research Group of Respiratory Diseases in Primary Care (IDIAP) also has professionals that provide methodological support to this project. Both Research Institutes (Sant Pau and IDIAP Jordi Gol i Gurina) also provide the support needed to carry out this study.

Experience of the Research Team

This project is coordinated by the Sleep Unit of Hospital de la Santa Creu i Sant Pau. In recent years, the research activity of this Unit has been focused on the study of clinical aspects related to sleep respiratory disturbances (SRD). The three Sleep Units involved in this project work under the *Spanish Sleep and Breathing Group* and are participating in several multicenter studies evaluating new methods of study the SRD and their clinical consequences. These studies have been published in high impact journals and have had a direct effect on the development of clinical practice guidelines.

The Sleep Unit of Hospital de Sant Pau is part of the *CIBER of Respiratory Diseases*.

One aspect that gives value and feasibility to this project is the inclusion of distinguished Primary Care professionals. Most of the Primary Care professionals involved are part of the Research Group of Respiratory Diseases in Primary Care (IDIAP). The group is composed of professionals from different Primary Care centers in the area called “Dreta de l'Eixample” in Barcelona. Their main objective is to standardize and coordinate the assessment of this disease at every health-level in order to achieve a proper use of all the available resources.

Practical applicability of the results

Nowadays, Primary Care needs to play a more important role in respiratory sleep disorders, not only by improving the level of clinical suspicion, but also by monitoring patients previously diagnosed and under treatment.

The incorporation of a clinical screening model and the validation of ambulatory monitoring studies at the Primary Care level would mean an improvement in the management of patients with OSA. This could provide greater flexibility in the diagnostic process and a decrease in waiting times. This screening model tries to save hospital visits and unnecessary tests, thereby avoiding the waste of resources. This would reduce the costs related to the disease.
